# Parental knowledge, attitudes, and practices related to school children nutrition in urban settings: cross-sectional study from Central Kazakhstan

**DOI:** 10.3389/fnut.2026.1784217

**Published:** 2026-03-27

**Authors:** Svetlana Rogova, Olga Plotnikova, Zhanerke Bolatova, Karina Nukeshtayeva, Olzhas Zhamantayev, Denis Turchaninov

**Affiliations:** 1 School of Public Health, Karaganda Medical University, Karaganda, Kazakhstan; 2 Department of Occupational Hygiene and Occupational Pathology, Omsk State Medical University, Omsk, Russia; 3 Department of Hygiene and Human Nutrition, Omsk State Medical University, Omsk, Russia

**Keywords:** attitudes and practices, knowledge, literacy, nutrition, parents

## Abstract

**Introduction:**

In urban settings, the relationship between parents' nutrition knowledge and children's actual dietary intake is often unstable, limiting the applicability of the linear interpretation of the Knowledge-Attitudes-Practices (KAP) model. The aim of this study was to assess how family sociodemographic characteristics and neighborhood characteristics are associated with parental KAP components and the organization of children's meals at school in Karaganda, Kazakhstan.

**Methods:**

A cross-sectional study was conducted using a stratified cluster sample. A total of 863 parents participated. The parent survey assessed knowledge about healthy eating (*K*), attitudes toward school meals (*A*), and school meal management practices (*P*). Linear and logistic regression models were used to analyze the associations between parental age, neighborhood, education level, and food expenditures with KAP indicators.

**Results:**

Parents' knowledge level was not statistically significantly associated with the organization of children's meals at school, indicating a gap between knowledge and practice. The most pronounced associations were observed with contextual factors. Children of parents under 30 years of age were more likely to eat in the school canteen (*OR* = 1.82; *p* < 0.05), while parents over 40 years of age were less likely to use it (*OR* = 0.32; *p* < 0.001) with a simultaneous increase in the likelihood of consuming sweets (*OR* = 1.34; *p* < 0.05). Living in the South-East district was associated with more frequent food purchases by children outside of school (*OR* = 1.87; *p* < 0.001) with a lower likelihood of bringing fast food from home (*OR* = 0.20; *p* < 0.001).

**Conclusion:**

The limitations of strategies focused solely on education and highlights the need for comprehensive measures that combine the modernization of school meals with the regulation of the food environment around schools.

## 1 Introduction

The global rise in diet-related non-communicable diseases (NCDs) represents one of the most serious and persistent threats to public health. This trend has become particularly pronounced among children and adolescents, where the prevalence of overweight and obesity has approached global epidemic levels in recent decades, significantly in-creasing the likelihood of developing chronic diseases in later life (1–3).

The most rapid increase in NCDs is observed in countries with transition economies, where demographic processes, intensive urbanization, and the transformation of food systems are leading to rapid changes in the dietary habits of the population (4, 5). In such conditions, in addition to already known risk factors, such as insufficient consumption of fruits and vegetables and excess caloric intake, additional threats are aggravated by urbanization: the high availability of ultra-processed foods, their active marketing promotion, and the large number of fast-food establishments within walking distance of residential areas. Kazakhstan is one example of such a dynamic change in context: according to projections by the World Obesity Federation, by 2030, more than 500,000 children in the country may be obese (5–7). These trends highlight the need for a comprehensive study of the determinants of family eating behavior in an urbanized environment.

Parents play a central role in shaping children's diets, acting as intermediaries between the external food environment and everyday food choices. They make grocery shopping decisions, determine the structure of the home diet, shape eating habits, and establish behavior patterns that children learn at an early age. However, parents' ability to fulfill this role is significantly challenged by changes in the urban environment (8–10).

Modern urban areas are characterized by a high concentration of fast-food outlets, the availability of high-energy foods, the active digitalization of marketing, and a significant amount of contradictory or inaccurate nutritional information (11, 12). Of particular concern is the rapid growth in the consumption of ultra-processed foods, which are increasingly displacing balanced diets. In such circumstances, parents need not only to possess basic knowledge about balanced nutrition but also to be able to critically assess marketing strategies and consciously create an accessible food environment for children (13–15).

Parental nutrition literacy is a key factor influencing children's eating behavior. High levels of knowledge about the principles of healthy eating contribute to a more varied diet, reduced consumption of high-energy foods, and the development of sustainable healthy eating habits (16–19). Conversely, low parental awareness is associated with irregular meal times, frequent fast-food consumption, and the risk of micronutrient deficiencies (20, 21). However, even knowledge does not guarantee its implementation in practice. Numerous studies demonstrate a gap between what parents know and how they act in real life, which is often due to the limitations of the urban environment, misconceptions about the health benefits of certain foods, or a lack of skills in critically evaluating nutritional information (22–25).

Despite growing interest in research into the factors that determine children's nutrition, data on the role of parents in Central Asian countries, including Kazakhstan, are extremely limited. National reports document low consumption of fruits and vegetables, rising obesity rates, and regional differences in food availability, but they do not address the mechanisms by which these outcomes are shaped at the family level. This gap is particularly pronounced in large urban centers, where socioeconomic contrasts, uneven food infrastructure, and a high density of fast-food outlets create a fragmented food environment (26–29).

Karaganda, one of the most industrialized and dynamically developing cities in Kazakhstan, is a model example of this type of environment. The city is characterized by significant inter-district differences in infrastructure, income levels, and access to healthy foods, making it a relevant model for studying the impact of urbanization on family eating behavior (28, 30, 31).

In this context, analysis of the Knowledge, Attitudes, Practices (KAP) profile of parents is a key tool for understanding how the urban environment transforms family behavior. The KAP model, recommended by the WHO, allows for the simultaneous assessment of cognitive and motivational factors, as well as real-life practices that influence product choice (32, 33). In the context of a highly informational and advertising-intensive urban environment, an integrative analysis of knowledge, attitudes, and practices makes it possible to identify behavioral determinants that remain invisible when using narrower analytical approaches. Meanwhile, most studies of KAP profiles have been conducted in countries with different cultural and socioeconomic characteristics (23, 32, 34), which emphasizes the need for regionally specific research in Kazakhstan. These indicators are limited in most universal KAP questionnaires on parental nutrition and, as a rule, are not identified as a separate school module, which limits their applicability within our design. Therefore, we used a proprietary questionnaire structured according to the KAP logic, where components *A* and *P* were treated as a set of individual indicators of the school context. In this study, the KAP model is not considered as a linear causal sequence of “knowledge–attitudes–practices.” It is used as a conceptual framework to organize indicators across three levels: cognitive (*K*), evaluative-motivational (*A*), and behavioral (*P*). The interpretation of the results is based on a socio-ecological approach, according to which the relationship between parental knowledge and children's nutrition practices in the school environment is determined not only by individual factors but also by the conditions of the school food environment, food availability around the school, intra-city differences, and family resource constraints.

Based on the identified gap, the aim of this study was to assess the KAP profile of parents of schoolchildren in Karaganda, including an analysis of the level of knowledge, attitudes and practices.

## 2 Materials and methods

A cross-sectional study was conducted to assess the knowledge, attitudes, and practices (KAP) of schoolchildren's parents regarding healthy eating and analyze their relationship with sociodemographic factors and the school food environment. Data were collected from September to November 2025. The study population consisted of parents (legal guardians) of students in grades 1–11 in public comprehensive schools in Karaganda, Kazakhstan. To ensure representativeness in the context of intracity heterogeneity, stratified cluster sampling was used.

Stratification was conducted by administrative district. Two contrasting districts were specifically selected: South-East and Maikuduk. These districts were chosen to allow them to be considered as contrasting inner-city territories with different characteristics of the residential environment and infrastructure development, allowing for an analysis of the variability of the studied indicators while maintaining citywide conditions. Maikuduk is characterized primarily by Soviet-era buildings, a historical connection to the city's industrial development, and a high proportion of standard housing. South-East, by contrast, belongs to later stages of urban expansion and is distinguished by a pre-dominance of modern high-rise residential buildings and a more developed retail and service infrastructure. The selection of districts was based on official urban planning and historical sources, reflecting differences in the stages of development, residential development types, and infrastructural development of the urban environment. These sources were used to contextualize the districts within the framework of an intracity comparative analysis (35–37).

In each of the selected districts, six comprehensive schools were selected (12 clusters in total), ensuring diversity in size, location within the district, and socioeconomic background. School administrations facilitated access to parents through official communication channels.

The minimum sample size was calculated using the Cochran formula for estimating the proportion of a characteristic in the general population: *n*0 = [*Z*^2^ × *p* × (1 – *p*)]/*ME*^2^, where *Z* = 1.96 (for 95% confidence level), *p* = 0.5 (maximum variability assumption), *ME* = 0.05 (permissible error). This calculation gave the base volume *n*0 = 384.

To account for the cluster design, a design effect correction (DEFF) was applied, calculated as: DEFF = 1 + (*m* – 1) × ρ, where *m*—average expected cluster size (taken as 60 respondents per school), ρ—intraclass correlation coefficient (ICC). Due to the lack of local data on ICC for similar KAP studies, a conservative estimate of ρ = 0.02 was used, which corresponds to the lower limit of values typical for socio-hygienic studies in educational institutions (38). Thus, DEFF = 1 + (60 - 1) × 0.02 = 2.18.

The adjusted minimum sample size was: *n*_adj = *n*0 × DEFF = 384 × 2.18 ≈ 837. Taking into account the possible percentage of incomplete questionnaires (~3%), the target sample size was set at 863 people.

The final analysis included 863 parents: 654 women (75.8%) and 209 men (24.2%). Participants were stratified by administrative district of residence (South-East: *n* = 466; Maikuduk: *n* = 397), educational level (higher: *n* = 561; secondary and secondary vocational: *n* = 302), and age (under 30: *n* = 276; 31–40: *n* = 334; over 40: *n* = 253).

The data collection instrument was an original structured questionnaire developed based on the Knowledge-Attitudes-Practices (KAP) model. Content validity was ensured by ensuring that the questions met the study objectives. To assess the clarity of wording, ease of navigation, and completion time, a pilot test was conducted on a separate sample of 35 parents not included in the main study. Based on the pilot test results, minor editorial revisions were made to the wording of several questions. The internal consistency (reliability) of the knowledge scale, calculated using Cronbach's alpha, was α = 0.76, indicating acceptable reliability of the instrument.

The final version of the questionnaire included four main sections:

1. Sociodemographic characteristics: age, gender, education, region of residence, and estimated monthly family food expenses. Parental age was categorized into three groups: < 30 years, 30–40 years, and >40 years. Parents younger than 30 years generally represent early parenthood, a stage characterized by limited parenting experience and greater reliance on recently acquired information sources, including digital media. The 30–40-year group corresponds to the most typical age range of parents of school-aged children in Kazakhstan and reflects a period of stabilized household routines and established dietary habits. Parents older than 40 years represent later parenthood, which is more often associated with stable long-term behavioral patterns and more traditional nutrition practices.
2. Knowledge of principles of healthy eating (*K*): 10 questions assessing awareness of principles of healthy eating, the functions of nutrients, nutrition-related diseases, and the ability to critically evaluate common food myths. Each question with one correct answer was worth 1 point, while questions with multiple correct answers were worth 2 points (provided all correct answers were selected according to the answer key). Thus, the maximum total score was 12. Based on this score, each respondent's knowledge level was classified as follows: low (1–5 points), moderate (6–9 points), or high (10–12 points).
3. Attitudes (*A*): The Attitudes component in this study reflected parents' perceptions and preferences regarding the school food environment and potential areas for improvement. It was assessed using questions aimed at identifying measures parents believed could contribute to improving their children's nutrition at school (improving the menu, updating the kitchen equipment, involving parents, increasing the competence of cafeteria staff, and educating children). Component A reflected parents' subjective assessments and expectations regarding the school food environment and was not a formal assessment of the school food policy (documents and regulations).
4. Practices (*P*): Questions assessing school-related practices: the child's attendance at the cafeteria, the nature of food brought from home, and after-school snacks. Component *P* reflected school-oriented nutrition practices of the child as reported by parents (self-report) and is additionally reflected in the Limitations section, which states that the analysis of official school documents and regulations was not included in the objectives of the study.

The questionnaire consisted of closed-ended questions with fixed answer options, including multiple choice and “don't know” options. The average time to complete was 10–12 min.

Data was collected using the Google Form platform. A link to the anonymous questionnaire was distributed to parents through the administrations and homeroom teachers of the selected schools. The first page of the online form contained full information about the study's objectives, confidentiality, and voluntary participation. Participants could proceed to the questionnaire only after confirming informed consent by activating the appropriate field.

The study protocol was reviewed and approved by the Local Ethics Committee of the Non-Commercial Joint-Stock Company “Karaganda Medical University” (extract from protocol No. 16 dated September 16, 2025). All procedures were conducted in accordance with the ethical standards set forth in the Declaration of Helsinki.

Descriptive statistics were used to summarize categorical variables as frequencies and percentages, and continuous variables as means and standard deviations. Associations between sociodemographic characteristics and the knowledge score (*K*) were assessed using multiple linear regression, while relationships with attitudinal preferences (*A*) and child feeding practices (*P*) were examined using binary logistic regression models. Although schools were used as recruitment channels, the analysis was conducted at the individual (parent) level. No school-level variables were included in the models; therefore, multilevel modeling was not applied. The regression models were fitted assuming independent observations.

## 3 Results

The study included 863 parents; mothers constituted the majority of respondents-−654 (75.8%)—while fathers represented 24.2% of the sample (*n* = 209) (Table 1).

**Table 1:** Level of parents' knowledge about healthy eating depending on socio-demographic characteristics (*n* = 863).

Variable		*n* (%)	Parents' knowledge and ideas about healthy eating, mean ±SD	*p*-value
What is your relationship status to your child?	Father	209 (24.2)	4,83 ± 2,51	0.421
	Mother	654 (75.8)	4.91 ± 2.41	
Age (years)	Up to 30	271 (32.0)	4.60 ± 2.24	0.794
	30–40	334 (38.7)	4.96 ± 2.67	
	Over 40	253 (29.3)	5.14 ± 2.29	
School district	Maikuduk	397 (46.0)	4.59 ± 2.44	0.002
	South-East	466 (54.0)	5.15 ± 2.40	
Food expenses	–	43 (5.0)	4.91 ± 2.76	0.342
	195$-234$ per month	128 (14.8)	4.95 ± 2.54	
	234$-292$ per month	121 (14.0)	4.91 ± 2.64	
	292$-350$ per month	138 (16.0)	5.00 ± 2.59	
	350$-390$ per month	141 (16.3)	4.55 ± 2.15	
	More than 390$ per month	265 (30.7)	5.03 ± 2.30	
	Less than 195$ per month	27 (3.1)	4.44 ± 2.41	
Education	Higher	561 (65.0)	5.17 ± 2.51	0.044
	Secondary/specialized secondary	302 (35.0)	4.39 ± 2.20	

The respondents' children attended schools in two districts of the city: South-East (54%; *n* = 466) and Maikuduk (46%; *n* = 397), ensuring comparable territorial representation.

An analysis of socioeconomic characteristics revealed that the largest proportion of families spent more than 390$ per month on food (30.7%; *n* = 265). 61.1% of respondents reported spending between 195 and 350$ per month, while 3.1% of families (*n* = 27) spent less than 195$ per month on food.

In terms of education level, the majority of respondents had higher education −65.5% (*n* = 561), while 35% (*n* = 302) had secondary or specialized education.

The mean total knowledge score in the study sample (*n* = 863) was 4.89 ± 2.44 points, with a range from 0 to 12. There were no statistically significant differences in knowledge about healthy eating between mothers and fathers (*p* = 0.421), nor between age groups (*p* = 0.794) (Table 1). At the same time, the level of knowledge was significantly higher among parents of children studying in schools in the South-East compared to the Maikuduk district (*p* = 0.002). Statistically significant differences were also found by educational level (*p* = 0.044), indicating that parents with higher education had higher literacy compared to parents with secondary or vocational education. Differences in family food expenditure were not statistically significant (*p* = 0.164).

About 42.9% of parents indicated that their child regularly eats in the school cafeteria, 37.9% indicated that they do not always eat there, and 19.2% reported that their child does not eat in the school cafeteria (Table 2). The mean total knowledge scores in these groups ranged from 4.87 ± 2.33 to 5.19 ± 2.65 points. Almost a quarter of parents (23.1%) indicated that their child buys food outside of school, 44.7% said they do so sometimes, while 14.0% indicated that such purchases occur rarely, and 8.2% never. The mean knowledge scores of parents in these groups ranged from 4.77 ± 2.26 to 5.36 ± 2.90 points.

**Table 2:** Level of parents' knowledge about healthy eating depending on attitudes and practices (*n* = 863).

Variable			*n* (%)	Mean ±SD	*p*-value
Does your child eat in the school cafeteria?	Yes		370 (42.9)	5.00 ± 2.39	0.397
	Not always		327 (37.9)	4.87 ± 2.33	
	He doesn't eat in the cafeteria		166 (19.2)	5.19 ± 2.65	
Does your child buy food outside of school during breaks, before, or after school (e.g., fast food, chips, sweets)?	Yes		199 (23.1)	4.77 ± 2.26	0.290
	Sometimes		386 (44.7)	4.93 ± 2.28	
	Rarely		121 (14.0)	5.36 ± 2.90	
	Never		71 (8.2)	5.13 ± 2.72	
	I find it difficult to answer		86 (10.0)	5.13 ± 2.34	
What foods does your child bring to school?	Homemade food	No	689 (79.8)	4.91 ± 2.36	0.043
		Yes	174 (20.2)	5.27 ± 2.61	
	Sandwiches	No	516 (59.8)	4.96 ± 2.45	0.668
		Yes	347 (40.2)	5.04 ± 2.37	
	Fast food	No	669 (77.5)	4.95 ± 2.36	0.108
		Yes	194 (22.5)	5.12 ± 2.62	
	Chips/crackers	No	421 (48.8)	4.91 ± 2.32	0.284
		Yes	442 (51.2)	5.07 ± 2.51	
	Sweets, chocolate, candy	No	284 (32.9)	5.07 ± 2.53	0.063
		Yes	579 (67.1)	4.95 ± 2.36	
	Fruit	No	629 (72.9)	4.94 ± 2.44	0.630
		Yes	234 (27.1)	5.12 ± 2.36	
	Soda/sweet drinks	No	397 (46.0)	4.77 ± 2.27	0.049
		Yes	466 (54.0)	5.18 ± 2.53	
	I don't know	No	793 (91.9)	4.99 ± 2.43	0.782
		Yes	70 (8.1)	4.99 ± 2.33	
What measures do you think could help improve your child's nutrition at school?	Menu improvement	No	428 (49.6)	4.66 ± 2.26	0.101
		Yes	435 (50.4)	5.32 ± 2.53	
	Kitchen equipment upgrades	No	474 (54.9)	4.74 ± 2.31	0.077
		Yes	389 (45.1)	5.31 ± 2.52	
	Parental engagement	No	430 (49.9)	5.17 ± 2.44	0.932
		Yes	431 (50.1)	4.82 ± 2.40	
	Improving cafeteria staff competencies	No	536 (62.1)	5.06 ± 2.50	0.161
		Yes	327 (37.9)	4.87 ± 2.28	
	Child education	No	401 (46.5)	5.32 ± 2.60	0.004
		Yes	462 (53.5)	4.71 ± 2.22	

An analysis of the food children bring to school revealed that parents most often cited sweets, chocolate, and candy (67.1%), followed by home-cooked food (20.2%). Fruit was brought by 27.1% of children, and carbonated and sweetened beverages by 54.0%. Average parental knowledge scores for the selected food categories ranged from 4.77 to 5.27 points. Among the possible measures to improve children's nutrition at school, parents most often mentioned child education (53.5%) and menu improvements (50.4%). Updating kitchen equipment and parental involvement were chosen by approximately half of the respondents. Average parental knowledge scores for these categories ranged from 4.66 ± 2.26 to 5.32 ± 2.60 points.

No significant differences in parents' average knowledge were found based on whether their child ate in the school cafeteria (*p* = 0.397). Similarly, the frequency of children's purchases of food products outside of school (fast food, chips, and sweets) was not statistically significantly associated with parents' knowledge (*p* = 0.290).

When analyzing the food products children brought to school, statistically significant differences were found for the “home-cooked food” category: parents whose children brought home-cooked food had higher average knowledge scores than those whose children did not (*p* = 0.043).

A significant association was also found for consumption of carbonated and sugary beverages (*p* = 0.049). For the remaining food categories (sandwiches, fast food, chips/crackers, sweets, and fruit), differences in average knowledge did not reach statistical significance.

In the block of questions concerning measures to improve children's nutrition at school, a statistically significant difference in the level of parents' knowledge was noted only for the “education of children” option (*p* = 0.004).

Linear regression results showed that parents' knowledge of healthy eating was statistically significantly associated with age and place of residence (Table 3).

**Table 3:** Socio-demographic factors of knowledge of the principles of healthy eating (*K*) and parents' subjective assessments and expectations regarding the school food environment (*A*).

Variable	Knowledge of principles of healthy eating (*K*)	Suggested measures to improve a child's school nutrition (*A*)				
		Improving the menu	Updating the kitchen equipment	Involving parents	Increasing the competence of cafeteria staff	Educating children
Age						
Younger than 30	−0.38^*^	0.93	1.31	1.68	1.14	1.38
30–40 years	1.68	Ref.				
Older than 40	0.18	0.99	1.03	1.29	1.18	1.61^**^
Location	VIF = 1.02					
Maikuduk	Ref.					
South-East	0.51^**^	0.66^**^	0.36^***^	5.09^***^	2.01^***^	0.56^***^
Food costs	VIF = 1.01					
Less 195$ per month	−0.25	1.10	1.24	0.65	1.32	0.71
195$-234$ per month	0.24	1.49	0.88	0.44^*^	1.21	0.37^**^
234$-292$ per month	0.17	1.81	1.13	0.59	0.93	0.38^*^
292$-350$ per month	0.30	2.11^*^	0.91	0.46^*^	1.03	0.41^*^
350$-390$ per month	−0.22	1.41	0.79	0.49	0.91	0.59
More than 390$ per month	0.12	2.01^*^	0.89	0.37^**^	0.85	0.42^*^
Education	VIF = 1.02					
Higher	3.13	Ref.				
Middle school/middle professional	2.39	1.06	0.74^*^	1.16	0.96	1.06
Parent	VIF = 1.01					
Mother	0.05	1.07	0.85	1.37	0.93	1.03
Father	1.67	Ref.				

^*^p < 0.05; ^**^p < 0.01; ^***^p < 0.001.

Parents under 30 demonstrated lower knowledge compared to the reference group aged 30–40 (β = −0.38; *p* < 0.05). However, no statistically significant differences in knowledge were found for parents over 40.

Place of residence also had a significant impact: living in the South-East district was associated with higher knowledge of healthy eating compared to the Maikuduk district (β = 0.51; *p* < 0.01). Family food expenditure, parental education level, and parental gender did not show statistically significant associations with knowledge levels, indicating that parents' theoretical awareness is relatively independent of socioeconomic status.

Parents over 40 were statistically significantly more likely to support educating children as suggested measure to improve a child's school nutrition than parents aged 30–40 (*OR* = 1.61; *p* < 0.05). No significant differences in measure preferences were found for parents under 30. Residence in the South-East district was one of the strongest factors influencing the choice of measures to improve nutrition. Compared to Maikuduk, parents in the South-East: significantly more likely to support increasing the competence of cafeteria staff (*OR* = 5.09; *p* < 0.001), more likely to choose involving parents and educating children (*OR* = 2.01 and *OR* = 0.56; *p* < 0.05), and less likely to support measures improving the menu and updating kitchen equipment (*OR* = 0.66 and *OR* = 0.36; *p* < 0.05). Family food expenditures demonstrated a consistent association with preferences for measures. Compared with families with the lowest expenditures, parents in the medium and high expenditure groups were statistically significantly less likely to choose involving parents (OR from 0.44 to 0.37; *p* < 0.05) and significantly more likely to support educating children, especially in the groups with expenses of $234–$292 and more than $390 per month (*OR* = 4.41 and *OR* = 3.36; *p* < 0.01–0.001). Parents with secondary or secondary vocational education were less likely to support updating the kitchen equipment compared to parents with higher education (*OR* = 0.74; *p* < 0.05). Parent gender was not statistically significantly associated with the choice of any measures (Table 3).

Parental age was a significant factor associated with children's actual dietary pat-terns. Children of parents under 30 were more likely to eat in the school cafeteria (*OR* = 1.82; *p* < 0.05). Conversely, parents over 40 were significantly less likely to have their child eat in the school cafeteria (*OR* = 0.32; *p* < 0.001), and their children were more likely to bring sweets to school (*OR* = 1.34; *p* < 0.05). Living in the South-East District was associated with more pronounced and divergent dietary patterns: children were significantly more likely to buy food outside of school (*OR* = 1.87; *p* < 0.001) and significantly less likely to bring fast food (*OR* = 0.20; *p* < 0.001). However, differences in fruit and sweets consumption were not statistically significant. As family spending on food in-creased, systemic changes in children's eating behavior were observed: children from families with average spending ($195–$234 and $234–$292) were more likely to eat in the school cafeteria (*OR* = 2.22 and *OR* = 4.41; *p* < 0.05–0.001), the likelihood of bringing fast food to school was lower in some groups (*OR* = 0.35–0.55; *p* < 0.05), and in the $350–$390 spending group, there was a decreased likelihood of bringing sweets (*OR* = 0.48; *p* < 0.05). Children of parents with secondary or vocational education were more likely to buy food outside of school (*OR* = 1.79; *p* < 0.01) and less likely to bring home-cooked food (*OR* = 0.58; *p* < 0.05) (Table 4).

**Table 4:** Socio-demographic factors of knowledge of School-oriented nutrition practices of the child (*P*).

Variable	School-oriented nutrition practices of the child (*P*)					
	Child eat in school cafeteria	Child buy food outside	Child brings to school			
			Home food	Fast food	Fruits	Sweets
Age	VIF = 1.01					
Younger than 30	1.82^*^	0.78	0.74	1.00	0.96	1.25
30–40 years	Ref.					
Older than 40	0.32^***^	0.79	0.89	0.79	1.20	1.34^*^
Location						
Maikuduk	VIF = 1.02					
South-East	0.58^**^	1.87^***^	0.20^***^	1.02	0.90	1.09
Food costs						
Less 195$ per month	1.01	0.59	0.88	0.90	1.16	0.87
195$-234$ per month	2.22^*^	0.65	1.71	0.55	2.57^*^	0.78
234$-292$ per month	4.41^***^	0.35^*^	1.11	0.53	1.79	1.02
292$-350$ per month	2.99^**^	0.41	1.43	0.55	2.20	0.84
350$-390$ per month	1.92	0.56	1.05	0.48^*^	1.79	0.63
More than 390$ per month	3.36^**^	0.56	1.56	0.62	2.18	0.61
Education	VIF = 1.02					
Higher	Ref.					
Middle school/middle professional	1.37	1.79^**^	0.58^**^	0.93	1.05	1.05
Parent						
Mother	1.02	1.01	1.01	1.04	0.97	
Father	Ref.					

^*^p < 0.05; ^**^p < 0.01; ^***^p < 0.001.

Parent gender was not statistically significantly associated with any of the nutritional indicators. In all models, the VIF values were in the range of 1.01–1.02, indicating the absence of multicollinearity and the stability of the obtained estimates.

## 4 Discussion

The study showed that, in a large urbanized city, the classical Knowledge-Attitudes-Practices (KAP) model only partially explains differences in the organization of school-age children's meals. Parents' knowledge of healthy eating principles was not consistently associated with how children's meals were organized. This gap between knowledge of healthy eating and eating behavior has previously been demonstrated in studies conducted in urbanized populations of countries with transition economies, where eating behavior is primarily influenced by the food environment and the social organization of everyday life, rather than by awareness (39, 40). Our results challenge the linear logic of the classical KAP model, strengthening the arguments in favor of a socio-ecological approach. We demonstrate that contextual factors such as neighborhood, parental age, and family food budget are stronger predictors of children's actual eating behavior than parental awareness of healthy eating.

Comparable observations are also presented in the literature on urban samples from countries with transition economies. The intra-city differences identified in Karaganda are interpreted as a local characteristic, while the general conclusion about the limited stability of the knowledge-practice relationship in the urban school environment is consistent with data from other studies. Higher awareness is not necessarily accompanied by favorable dietary practices. For example, among adolescents in Tehran, the eating behavior did not correspond to the level of nutrition knowledge (40). A similar gap was described among parents in China, where the relationship between knowledge, practices, and attitudes was minimal, and the attitude-practice relationship was weak, indicating a limited explanatory role of the cognitive component without taking into account environmental factors (23). In Singapore, inconsistency in KAP profiles, including a discrepancy between knowledge and practices, was also noted (25). Furthermore, studies of the urban food environment show that food choice is largely associated with the availability and characteristics of the environment and household resource constraints (11, 12). Taken together, these data are consistent with our interpretation: in urban school settings, parental knowledge may not translate into sustainable practices without considering the context in which everyday food decisions are made.

It is known that the relationship between nutrition knowledge and actual behavior in children and adolescents is often weak or unstable (41, 42), and in urban populations, moderate knowledge can coexist with unfavorable dietary patterns (39, 40, 43). Our data not only confirm this gap but also reveal its possible structural cause in the context of urbanization. We found that the level of parental knowledge did not vary depending on education, indicating its leveling as a social marker. Thus, in an urban environment, basic knowledge about healthy eating appears to be transformed into a widely accessible but theoretically “inert” resource that loses its ability to differentiate social groups. This explains why the cognitive component of the KAP model loses its explanatory power: being equally prevalent across different social groups, it ceases to differentiate families and, therefore, cannot predict differences in their school meal planning practices. In the bivariate analysis, parental knowledge differed according to education level. However, after adjustment for sociodemographic factors in the multivariable model, this association was no longer statistically significant. This suggests that in an urban environment, basic knowledge about healthy eating may be relatively widespread across social groups, although crude differences between education categories can be observed before accounting for confounders.

This mechanism is clearly illustrated by the example of parents' age. Parental age shapes children's eating behavior through lifestyle, not through knowledge. Children of young parents (under 30) eat in the cafeteria more often, reflecting a strategy of dele-gating meals to school due to busy schedules and time constraints (44). Conversely, children of parents over 40 use the cafeteria less often and are more likely to bring sweets. This indicates an autonomous family model, where personal preferences and control prevail over the school routine (45). Thus, age acts as a marker of the type of family interaction with the environment: reliance on external institutions or internal traditions. In both cases, behavior is shaped bypassing the cognitive component, demonstrating the limitations of the linear logic of the KAP model in an urban context.

Intracity differences in schoolchildren's nutrition are largely determined by the local food environment. The presence of a developed retail infrastructure near schools creates alternatives to family strategies, reducing the role of parental decisions even when they are sufficiently informed. Spatial accessibility of food products becomes a competing factor, as supported by data on the influence of the food environment on food choices (46) and the direct link between the availability of unhealthy food near schools and its consumption, regardless of the family context (47). Thus, the neighborhood of residence acts not simply as a backdrop but as an active determinant that modifies or limits the implementation of parental intentions regarding their child's nutrition.

Unlike knowledge, family economic resources proved to be a reliable predictor of nutrition practices. Increased food expenditures were associated with more frequent visits to the school cafeteria and a reduced likelihood of bringing fast food and sweets. This confirms data showing that financial security directly determines dietary patterns and the availability of organized meal options, in contrast to educational measures that ignore financial barriers (42). School feeding programmes thus serve an important **equalizing** function by providing a stable source of food, which is particularly important for children from less affluent families and helps to reduce socio-economic inequalities in nutrition (48).

An analysis of parental attitudes reveals a shift in responsibility toward the school. Priority measures are seen as improvements at the institutional level: menu quality, dining conditions, and staff competence. Measures requiring active behavioral involvement from families are less clearly supported and depend on the context. This distribution of preferences reflects a perception of the school as the primary agency responsible for school-age nutrition. Parents focus on “manageable” changes in the system, while behavioral initiatives requiring personal resources and involvement receive variable support. This is consistent with data showing that educational components are most effective within comprehensive programs that combine education with changes in the environment and nutritional organization (49, 50).

Taken together, obtained results allow us to conclude that in urban settings, the link between parental knowledge and children's eating practices is disrupted by con-textual influences. Our study expands our understanding of this gap, demonstrating that it is not simply a “knowledge deficit” but a systemic consequence of urbanization: basic knowledge becomes a widely accessible but inert resource, having lost its power to differentiate social groups.

From a theoretical perspective, this means that the “knowledge-practice” relationship in the city is fundamentally mediated by the availability of alternative food options, family economic resources, and the organization of school meals. This conclusion supports and concretizes the socio-ecological approach (46, 51), which requires analyzing individual factors inextricably linked to environmental and institutional conditions (12, 44, 45, 52).

The practical implication is the need to shift the focus of school nutrition policy. Educational measures aimed solely at raising awareness (41, 42) will have a limited effect. Priority should be given to structural interventions: strengthening the role of school cafeterias as an accessible and attractive standard of nutrition (48) and regulating the food environment near schools to reduce the availability of unhealthy alternatives (12, 47, 52). This comprehensive approach, focusing on changing the conditions of choice, is most consistent with both the data of our study and the expectations of the parents themselves (50, 51).

The study has several limitations. Its cross-sectional design does not allow for establishing causal relationships and the findings should be interpreted as associations rather than causal effects. The data are based on parental self-reports, which may introduce bias. Furthermore, the study was conducted in a single urban center, limiting the generalizability of the results. The Attitudes and Practices components were considered a set of contextual indicators of the school environment and everyday school practices, rather than independent psychometric scales. The Attitudes component reflected parental assessments of the school food environment and did not include an analysis of official school food policy (documents/regulations). The Practices component relies on parental self-reports of the child's behavior, which is subject to measurement error and requires caution in data interpretation. In addition, the attitude and practice domains were measured using context-specific indicators rather than standardized psychometric scales; although the questionnaire underwent expert review and pilot testing, the absence of external psychometric validation may introduce measurement error and limits direct comparability with studies using validated KAP instruments. Because clustering at the school level was not explicitly modeled, standard errors may be slightly underestimated. Therefore, statistically significant associations should be interpreted with caution. However, a strength of the study is its detailed analysis of school meal arrangements and consideration of intra-city heterogeneity, which allowed us to identify the influence of the local context. Future research should include longitudinal designs, incorporate objective assessments of the food environment (e.g., audits of retail outlets near schools), and replicate the study in other urban regions.

## 5 Conclusions

This study, conducted among parents of schoolchildren in Karaganda, Kazakhstan, revealed that in an urbanized environment, school meal planning is more dependent on sociodemographic characteristics and family lifestyle than on parents' knowledge of healthy eating. These findings highlight the limited effectiveness of strategies focused solely on education and underscore the need for comprehensive measures, including modernizing school meals and improving the availability of healthy foods near schools. These findings have important implications for the development and implementation of effective strategies to optimize school meal programs and create a supportive food environment for children in Kazakhstan and other urbanized regions.

## Data Availability

The raw data supporting the conclusions of this article will be made available by the authors, without undue reservation.
